# Like sheep, like humans? Right ventricular remodelling in a preterm‐born ovine model

**DOI:** 10.1113/JP276067

**Published:** 2018-05-10

**Authors:** Afifah Mohamed, Paul Leeson, Adam J. Lewandowski

**Affiliations:** ^1^ Oxford Cardiovascular Clinical Research Facility, Division of Cardiovascular Medicine, Radcliffe Department of Medicine University of Oxford Oxford UK

**Keywords:** preterm birth, cardiomyocytes, heart development, right ventricle

Preterm birth affects more than 10% of births worldwide, with over 80% of these individuals born moderately preterm; between 32–36 weeks’ gestation. Experimental and human studies have shown that preterm birth associates with structural remodelling of the heart (Bensley *et al*. 2010; Bertagnolli *et al*. 2014), with human studies in early adulthood demonstrating the extent of remodelling and reductions in systolic function to be greatest in the right ventricle (RV) (Lewandowski *et al*. 2013). Preterm birth has recently been identified as a novel risk factor for early heart failure (Carr *et al*. 2017), and is characterized by an impaired myocardial functional reserve in young adulthood under physiological stress (Huckstep *et al*. 2018). There is thus a need for experimental and human studies investigating the underlying mechanisms leading to preterm cardiac alterations.

In this issue of *The Journal of Physiology*, Mrocki *et al*. (2018) assessed the RV dimensions and function in preterm‐born sheep using ultrasound and tissue characterization of excised, perfusion‐fixed hearts. The authors studied male sheep delivered moderately preterm (*n* = 7; 132 ± 1 days, equivalent to 36 weeks’ gestation in humans) or at term (*n* = 7; 147 ± 1 days, equivalent to 40 weeks’ gestation in humans) and followed them up to 14.5 months of age; equivalent to 20 years of age in humans. Their results showed that preterm‐born sheep had thinner RV walls, a higher reserve of immature undifferentiated cardiomyocytes, lower cardiomyocyte numbers and smaller cardiomyocyte areas. Pulmonary artery peak systolic blood flow velocities were lower in the preterm‐born sheep, which may be due to a reduced RV contractile capacity. Together, these findings signify reductions in RV functional and adaptive capacity in preterm‐born adult sheep.

The research group has previously investigated RV changes in a similar model at 9 weeks after term‐equivalent age (equivalent to childhood in humans) (Bensley *et al*. 2010). Contrary to the current study, they showed that preterm‐born sheep had increased cardiomyocyte volumes, no difference in cardiomyocyte cell number, and a 6.6‐fold increase in collagen content in the RV compared to term‐born sheep. Furthermore, although not significant, heart weight of preterm‐born sheep was increased. These differences in findings between the two ages in sheep may be the result of preterm birth leading to altered development over time. Nevertheless, sheep studied at 14.5 months received higher antenatal glucocorticoid doses than sheep in their previous study (Bensley *et al*. 2010). This may cause greater disruption in hyperplasia and alter cell number long‐term. Indeed, compared with their findings at 9 weeks after term‐equivalent age, there was only a 3‐fold increase in RV cardiomyocyte number in preterm‐born sheep at 14.5 months *versus* a 5.1‐fold increase in the term‐born sheep. Whether previous findings at 9 weeks after term‐equivalent age are repeated when higher doses of antenatal glucocorticoids are used is of interest.

Our previous cardiovascular magnetic resonance study in young adult humans showed reductions in RV stroke volume, end‐diastolic volume, and ejection fraction (Lewandowski *et al*. 2013). Although RV stroke volume relative to weight was 12% lower in the preterm‐born sheep in the study by Mrocki *et al*. (2018) at 14.5 months, this was not significant. This lack of significance could reflect the small sample size and large variability in measurement of the RV, which has a complex geometry. Alternatively, this may reflect the differences in gestational age at birth between human and ovine studies, given only 2% of the human cohort was born at 36 weeks’ gestation (Lewandowski *et al*. 2013); the equivalent gestational age of the preterm‐born sheep. In addition, human preterm birth is not simply an abrupt termination of pregnancy, but instead it is typically a pathophysiological event, often characterized by a heightened inflammatory state. This may have direct relevance to structural remodelling of the heart.

The postnatal environment may also differ in humans. One of the strengths of the ovine model presented by the authors is that it limits exposure to the many confounding factors associated with studies in humans, and therefore dissimilarities in findings are not necessarily due to species differences but may relate to the many confounding lifestyle factors influencing growth of the human heart. In preterm‐born humans, blood pressure is increased as early as childhood and it is possible that the increased arterial pressure acts as a greater hypertrophic stimulus for the immature, undifferentiated cardiomyocytes in preterm‐born individuals. Arterial pressure was not increased in preterm‐born sheep at 14.5 months in the current study and further work may be needed for optimization of animal model physiology to replicate the human condition. These may then allow exploration of the relevance of blood pressure modification to triggering, or prevention, of cardiac remodelling in those born preterm. Lifestyle interventions in humans to manage blood pressure throughout development may be especially important for prevention of increased cardiac mass.

Given the large proportion of the population born preterm, further experimental work to better understand cardiac remodelling as a result of preterm birth is of mechanistic and public health importance. The pathophysiology of preterm birth, perinatal interventions and heterogeneity of postnatal lifestyle exposures makes direct comparison of preterm‐born humans and sheep challenging but offers an opportunity for further development of both human and experimental studies. If specific pathways leading to cardiac alterations are identified experimentally, it may be possible to translate these findings to human clinical interventions to reduce cardiovascular disease risk in preterm‐born individuals.

## Additional information

### Competing interests

The authors have no conflicts of interest.

### Funding

Dr Adam Lewandowski is funded by a British Heart Foundation Intermediate Research Fellowship (FS/18/3/33292). Ms Afifah Mohamed is funded by the Malaysian Government and the National University of Malaysia.
